# Upper limb function in Duchenne muscular dystrophy: 24 month longitudinal data

**DOI:** 10.1371/journal.pone.0199223

**Published:** 2018-06-20

**Authors:** Marika Pane, Giorgia Coratti, Claudia Brogna, Elena Stacy Mazzone, Anna Mayhew, Lavinia Fanelli, Sonia Messina, Adele D’Amico, Michela Catteruccia, Marianna Scutifero, Silvia Frosini, Valentina Lanzillotta, Giulia Colia, Filippo Cavallaro, Enrica Rolle, Roberto De Sanctis, Nicola Forcina, Roberta Petillo, Andrea Barp, Alice Gardani, Antonella Pini, Giulia Monaco, Maria Grazia D’Angelo, Riccardo Zanin, Gian Luca Vita, Claudio Bruno, Tiziana Mongini, Federica Ricci, Elena Pegoraro, Luca Bello, Angela Berardinelli, Roberta Battini, Valeria Sansone, Emilio Albamonte, Giovanni Baranello, Enrico Bertini, Luisa Politano, Maria Pia Sormani, Eugenio Mercuri

**Affiliations:** 1 Pediatric Neurology and Nemo Clinical Centre, Fondazione Policlinico Universitario "A. Gemelli IRCSS", Università Cattolica del Sacro Cuore, Rome, Italy; 2 John Walton Muscular Dystrophy Research Centre, Institute of Genetic Medicine, International Centre for Life, Newcastle Upon Tyne, United Kingdom; 3 Department of Clinical and Experimental Medicine and Nemo Sud Clinical Center, University of Messina, Messina, Italy; 4 Unit of Neuromuscular and Neurodegenerative Disorders, Department of Neurosciences, Bambino Gesù Children’s Hospital, Rome, Italy; 5 Cardiomiology and Medical Genetics, Department of Experimental Medicine, Second University of Naples, Naples, Italy; 6 Department of Developmental Neuroscience, IRCCS Stella Maris, Pisa, Italy; 7 Center of Myology and Neurodegenerative Disorders and Physical and Rehabilitation Medicine Unit, Istituto Giannina Gaslini, Genova, Italy; 8 Neuromuscular Center, AOU Città della Salute e della Scienza, University of Torino, Turin, Italy; 9 Department of Neurosciences, University of Padua, Padua, Italy; 10 Child and Adolescence Neurological Unit, National Neurological Institute Casimiro Mondino Foundation, IRCCS, Pavia, Italy; 11 Child Neurology and Psychiatry Unit, IRCCS Institute of Neurological Sciences, Bellaria Hospital, Bologna, Italy; 12 NeuroMuscular Unit IRCCS Eugenio Medea, Bosisio Parini, Italy; 13 Developmental Neurology, Neurological Institute Carlo Besta, Milan, Italy; 14 Centro Clinico Nemo, Milan, Italy; 15 Biostatistics Unit, Department of Health Sciences, University of Genoa, Genoa, Italy; University of Rome La Sapienza, ITALY

## Abstract

The aim of the study was to establish 24 month changes in upper limb function using a revised version of the performance of upper limb test (PUL 2.0) in a large cohort of ambulant and non-ambulant boys with Duchenne muscular dystrophy and to identify possible trajectories of progression. Of the 187 patients studied, 87 were ambulant (age range: 7–15.8 years), and 90 non-ambulant (age range: 9.08–24.78). The total scores changed significantly over time (p<0.001). Non-ambulant patients had lower total scores at baseline (mean 19.7) when compared to the ambulant ones (mean 38.4). They also had also a bigger decrease in total scores over 24 months compared to the ambulant boys (4.36 vs 2.07 points). Multivariate model analysis showed that the Performance of Upper Limb changes reflected the entry level and ambulation status, that were independently associated to the slope of Performance of Upper Limb changes. This information will be of help both in clinical practice and at the time of designing clinical trials.

## Introduction

In 2012 an international group of clinicians, physical therapists, patients, advocacy groups and industries worked together to develop the Performance of the Upper Limb (PUL), a functional scale specifically designed for assessing upper limb function in Duchenne muscular dystrophy (DMD) [1–3]. The PUL was designed with the aim of reflecting the proximal to distal progression of muscle weakness typically observed in DMD. It includes three domains (shoulder, mid- and distal), each including items exploring activities easily related to activities of daily living that both patients and clinicians identified as relevant. The scale was originally designed using Rasch analysis, a psychometric method that aimed to improve validity and reliability of the scale. [3]

The scale, in its version labelled 1.2, has been used for over three years and has proved to be reliable, reproducible and suitable for international multicentric settings [4]. It has also been found to be related to 6MWT in ambulant patients [5]. In non-ambulant DMD boys has also been found to be sensitive to changes depending on the steroid regime[6]. Since the original version of the PUL was published[4], following the acquisition of longitudinal data in a larger cohort, further clinical testing and Rasch analysis have suggested to modify the scale by deleting redundant items and simplifying the number of scoring, options for most items to 0, 1 or 2; shoulder level items, hands to mouth, tearing paper, trace path, push on light, supination, pick up coins, number diagram and pinch grip to 0 and 1. This aimed to improve the hierarchical response options of individual items within the context of all items. Other items, such as some shoulder items, moving and stacking light cans and moving heavy cans, key and three finger pinch items were deleted while one item—moving heavy can diagonally- was added. A revised scale, PUL 2.0, was therefore proposed with 22 items. The items in the two versions measure the same construct (PUL 1.2 and 2.0) but the scoring system is different as the PUL 2.0 has scoring options that vary across the scale between 0–1 to 0–2 according to performance as opposed to the PUL 1.2 that has a broader choice of scoring options (up to 0–5 in some items) but which were shown to lack clinical distinctiveness.

We report natural history data on a large cohort of DMD boys assessed with the PUL 2.0 for 24 months. The aim of the study was to establish the variability of the results over 24 months in both ambulant and non-ambulant DMD boys and to identify possible trajectories of progression.

## Materials and methods

The study is part of a longitudinal multicentric project aimed at establishing changes in upper limb function in DMD. Consent was obtained from both adults and parents or guardians of the minors included in the cohort, all clinical investigation were conducted according to the principles expressed in the Declaration of Helsinki. The study was approved by the Ethical Committees of all the participating centers (Catholic University of the Sacred Heart; University of Messina; Bambino Gesù Children’s Hospital; Second University of Naples; IRCCS Stella Maris; Istituto Giannina Gaslini; University of Torino; University of Padua; National Neurological Institute Casimiro Mondino Foundation; IRCCS Institute of Neurological Sciences, Bellaria Hospital; IRCCS Eugenio Medea; Neurological Institute Carlo Besta; Centro Clinico Nemo Milan). As part of this, all the Italian tertiary care centers for neuromuscular disorders consecutively enrolled all the DMD patients attending their routine follow up clinics between September 2012 and February 2014. The only exclusion criteria were related to the inability to perform the test because of severe cognitive or behavioral problems.

As in a previous cross sectional study [4] there was an increase of scores in the younger DMD boys due to a combination of development, increased height and hand size, with an obvious slope of deterioration after the age of 7 years, we only included boys > 7 years. Patients who entered clinical trials were also excluded.

### PUL 2.0

The PUL 2.0 includes (S1 figure) an entry item to define the broad starting functional level, and 22 items subdivided into shoulder level (6 items), mid- level (9 items) and distal level (7 items) dimension[3]. The entry items is based on a revised version of the Brooke score and ranges from score 0 –no useful hand function—to score 6 full shoulder abduction—no weakness. For weaker patients a low score on the entry item means high level items do not need to be performed as they would not be achieved.

Each dimension (shoulder, mid-, distal) can be scored separately.

The assessments were performed on forms that allowed to score for both PUL 1.2 and 2.0 but we only report the results for the new version (PUL 2.0).

In the PUL 2.0 there is a maximum score of 12 for the shoulder level, 17 for the mid- level, and 13 for the distal level. A total score can be achieved by adding the three level scores (max global score 42). Details of the training sessions and of the reliability studies have already been reported for the original PUL version[4]. New training sessions were performed for the new scale with similar level of agreement.

### Statistical analysis

Data were analyzed in two ways. First we calculated the PUL 2.0 changes at 12 and 24 months according to the entry level in both ambulant and non-ambulant DMD boys.

The relationship of the time course of PUL over 24 months, entry level and ambulatory status was then analysed using a mixed effect linear model with a random intercept. The model aims to answer the questions whether the PUL changes significantly over 24 months and whether the change is different according to entry level, age and ambulatory status (two level interaction) or according to a combination of these factors (three levels interaction).

The same model was applied to the shoulder, mid- and distal subscores.

## Results

One hundred and seventy-seven patients fulfilled the inclusion criteria, 87 were ambulant (age range: 7–15.8 years, mean: 9.54 (SD: 1.89), median: 9.33) and 90 non ambulant (age range: 9.08–24.78, mean: 16.42 (SD: 3.98), median: 16.25). One hundred and thirty-nine were on steroids and 38 (all non ambulant) were not.

### PUL 2.0

The total scores at baseline ranged between 3 and 42 (mean: 28.81 SD: 12.5). The 24 month changes of the total scores ranged between -21 and +7 (mean -3.23 SD 4.8).

**Fig 1:**
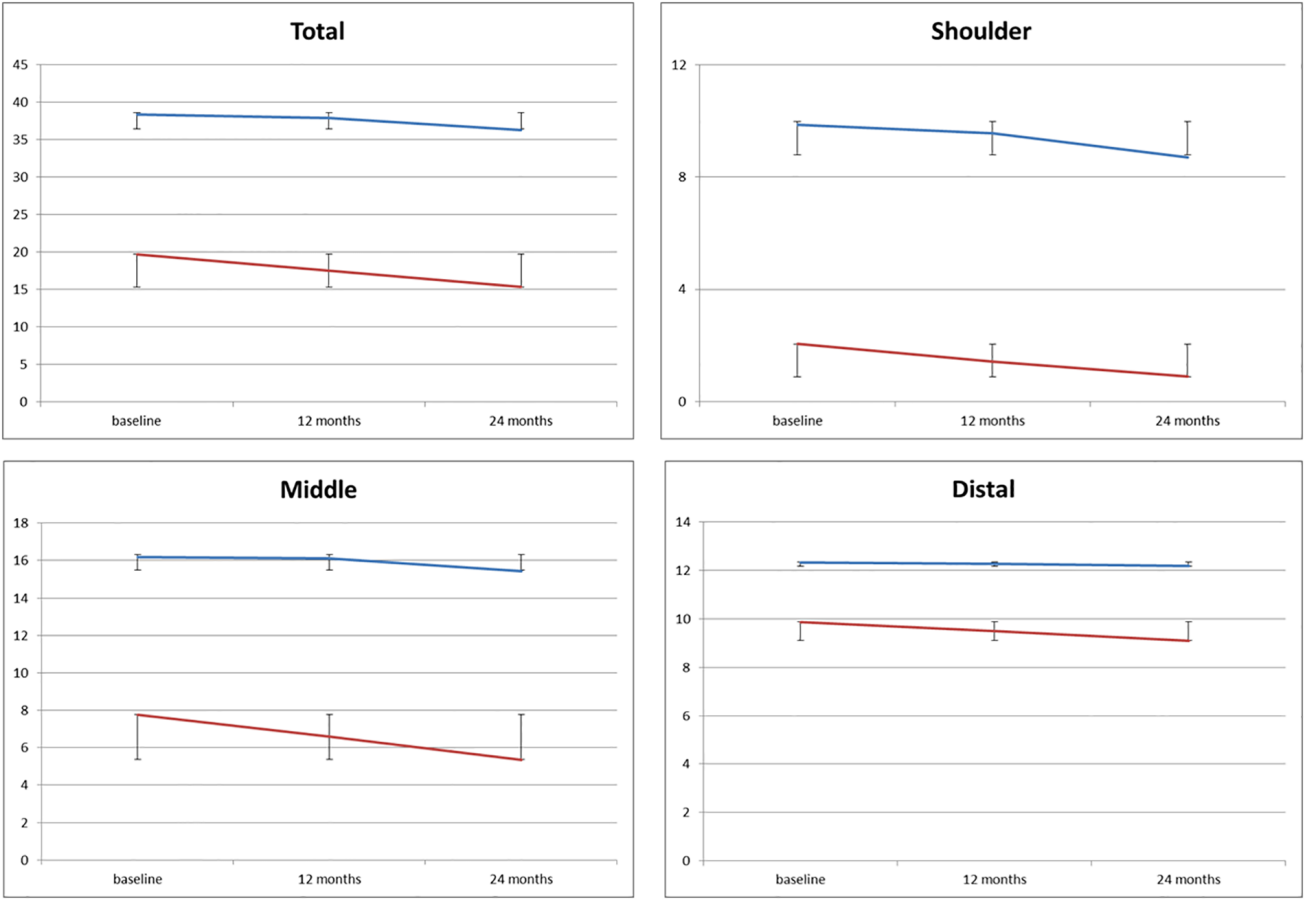
Mean changes in the total scores and in the three domains in ambulant and non ambulant patients.

Table 1 and Fig 1 report details of the 12 and 24 month PUL scores subdividing the cohort into ambulant and non-ambulant.

**Table 1:** Details of PUL2.0 scores in ambulant and non ambulant patients at baseline, 12 and 24 months.

PUL 2.0	Baseline		12 months		24 months	
**TOTAL SCORES**	**Range**	**Mean (SD)**	**Range**	**Mean (SD)**	**Range**	**Mean (SD)**
AMBULANT	24–42	38.38 (4.15)	23–42	37.93 (4.47)	19–42	36.31 (6.36)
NON AMBULANT	3–42	19.70 (10.92)	2–40	17.53 (10.05)	2–42	15.34 (8.96)
**SHOULDER SCORES**	**Range**	**Mean (SD)**	**Range**	**Mean (SD)**	**Range**	**Mean (SD)**
AMBULANT	3–12	9.86 (2.49)	2–12	9.57 (2.62)	2–12	8.71 (3.42)
NON AMBULANT	0–12	2.06 (3.68)	0–12	1.42 (3.14)	0–12	0.9 (2.57)
**MID- SCORES**	**Range**	**Mean (SD)**	**Range**	**Mean (SD)**	**Range**	**Mean (SD)**
AMBULANT	10–17	16.18 (1.48)	8–17	16.09 (1.79)	6–17	15.41 (2.77)
NON AMBULANT	0–17	7.77 (5.81)	0–17	6.61 (5.59)	0–17	5.355 (4.83)
**DISTAL SCORES**	**Range**	**Mean (SD)**	**Range**	**Mean (SD)**	**Range**	**Mean (SD)**
AMBULANT	9–13	12.33 (0.92)	10–13	12.26 (0.93)	10–13	12.18 (0.98)
NON AMBULANT	3–13	9.86 (2.55)	2–13	9.50 (2.59)	2–13	9.08 (2.82)

### Changes according to entry level

**Fig 2:**
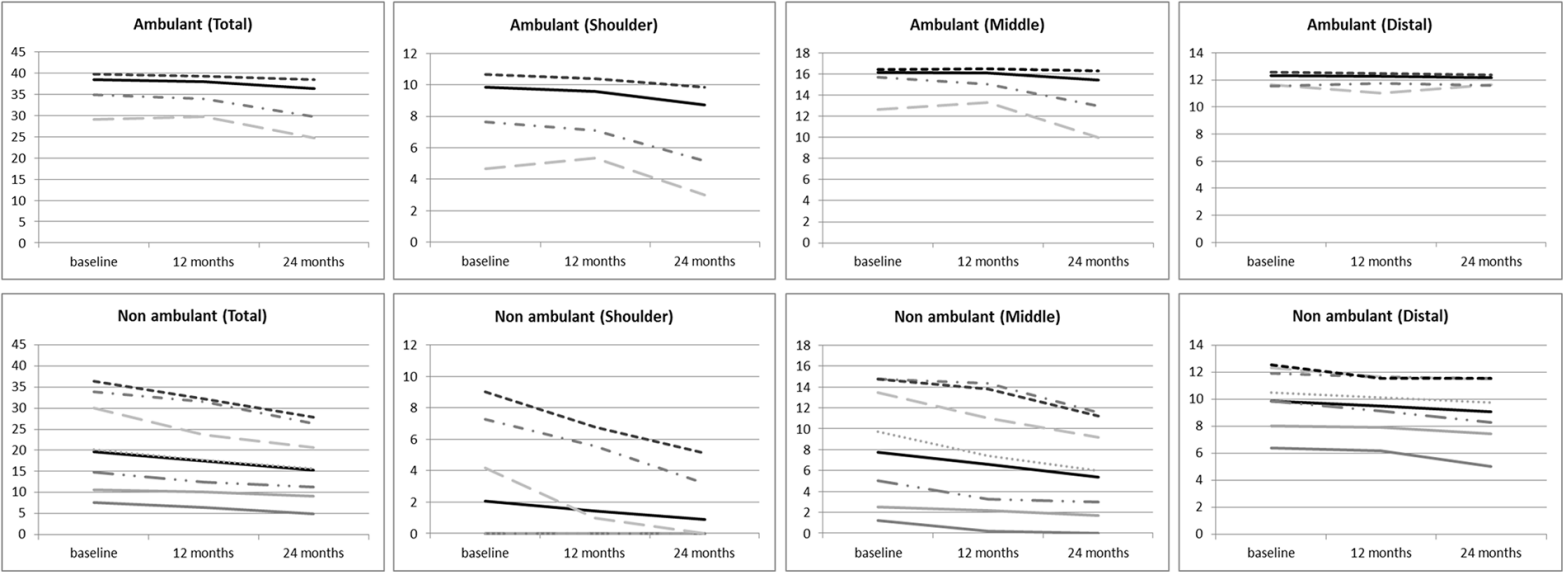
Progression of changes according to entry level scores.

In the subgroup of ambulant patients 67 of the 87 boys (77%) had an entry item of 6. Their mean24 month changes were -1.19 in the total PUL scores, of which -0.79 at shoulder level, -0.19 at mid- and -0.20 at distal level (Fig 2).

Twenty ambulant boys (23%) had an entry score of 4 or 5. Their mean 24 month changes were -5 points in the total PUL scores, of which -2.35 at shoulder level, -2.70 at mid- and 0.05 at distal level. None of the ambulant boys had scores lower than 4.

In the subgroup of non-ambulant patients 26 (20%) had an entry level between 6 and 4 and had the fastest decline. Their mean24 month changes were -8.30 in the total PUL scores of which -4.04 at shoulder level, -3.58 at mid- and -0.69 at distal level.

Twenty-eight (31%) had entry level scores of 3 and 2 and showed an intermediate decline. Their mean24 month changes were -4.25 in the total PUL scores, of which 0 at shoulder level, -3.32 at mid- and -0.93 at distal level.

Thirty-six patients (40%) had an entry level of 1 and 0 and showed less decline. Their mean24 month changes were -1.58 in the total PUL scores of which 0 at shoulder level, -0.86 at mid- and -0.72 at distal level. Fig 2 reports trajectories of progression based on their entry level scores.

Using the mixed effect linear model with a random intercept, entry level, age and ambulatory status were highly correlated. Ambulant children had a mean age of 9.5 years (SD = 1.9) and a mean entry level of 5.7 (SD = 0.5); non-ambulant children had a mean age of 16.4 years (SD = 4.0) and a mean entry level of 2.7 (SD = 1.8), (p<0.001 for both comparisons).

Overall PUL changed significantly over time (mean PUL yearly change -1.5 (SE = 0.18), p<0.001). The PUL change was significantly associated to entry level (children with entry level lower than 6 had 1.05 point loss per year more than children with entry level = 6, p = 0.003), age (children with age higher than the median value of 11.6 years had 0.75 point loss per year more than children with age lower than 11.6, p = 0.03) and ambulation status (non-ambulant children having 1.13 point loss per year more than ambulant children, p<0.001), when examining each factor separately. In a multivariate model, only entry level and ambulation status resulted independently associated to the slope of PUL change.

In a multivariate model including all the factors, age was no longer significant, while the ambulation status and the entry levels remains as independent predictors of PUL change over time. In particular, a 3 levels interaction (time by ambulation status by entry level) was significant (p = 0.04), indicating that the difference of time trend between ambulant and non-ambulant children was dependent on entry level.

## Discussion

Our 24 month longitudinal data obtained in a large cohort of DMD boys confirmed that the PUL 2.0 can detect the progression of upper limb involvement, as reported in our previous study in non-ambulant boys followed longitudinally for 12 months using the previous 1.2 version of the PUL [6]. In the present study, using the PUL 2.0 in a wider cohort, including both ambulant and non-ambulant, the PUL scores changed significantly over 24 months. The range of changes was quite wide but the variability could be partly reduced when the cohort was subdivided into ambulant and non-ambulant subgroups. The two subgroups had different baseline scores and different patterns of changes. Ambulant patients had much higher total scores at baseline when compared to the non-ambulant ones. This largely reflected the fact that they generally had full or near full scores at shoulder and mid- level that contributed significantly to the total score. In these patients, the PUL is often used in combination with other scales such as the 6 minute walk test. [5] In contrast, in the non-ambulant subgroup, only few of them had shoulder scores above 4, with the majority having a score of 0.

At mid- and distal level range and mean values were also much lower than in the ambulant boys but a floor effect could only be consistently observed in the shoulder domain in the non ambulant patients.

As expected, over 24 months non-ambulant patients showed a bigger decrease in total scores compared to the ambulant ones (4.36 vs 2.07 points). The pattern of decrease also largely reflected the baseline scores in the different domains. In the ambulant group the highest loss was at shoulder level while in the non-ambulant groups was at elbow level as the very low shoulder scores at baseline in the non-ambulant allowed only a limited decrease.

The variability of changes related to the baseline scores prompted us to further stratify the cohort according to their entry level item at baseline. This new analysis allowed us to identify more accurate trajectories of progression in both ambulant and non-ambulant patients.

In ambulant patients, the boys who could complete the entry items, with a score of 6, were overall stable, with small 24 month changes in the shoulder domain and little or no changes at mid- and distal level. Ambulant boys with an entry score of 4 or 5 in contrast showed some changes both at shoulder and mid- level. None of the ambulant boys had scores lower than 4.

The non-ambulant boys with an entry level between 4 and 6 had some residual scores at shoulder level, that were generally lost over the 24 month observation time, with also a rapid decrease at mid- level and an overall larger decrease when compared to the other subgroups.

At the other end of the spectrum, those with entry level scores of 0 and 1 already had low total scores at baseline, and having less activities to be lost, showed small decrease over time. Patients with intermediate entry level scores (2 and 3) had generally an intermediate decrease of the total scores mainly related to loss at mid- domain.

Overall, the PUL changes therefore reflected mainly the entry level and ambulation status, as also proved by multivariate model analysis that showed that both variables resulted independently associated to the slope of PUL change.

The results of our study are not easily comparable to our previous findings[6]. This is because the present study also includes ambulant patients and, more importantly, because of the different versions of the PUL used. While the items and the activities are almost identical in the two versions and aim to measure the same construct, the 2.0 version reported in this study has the advantage of a simplified scoring system which improves it measurement qualities. The new version has also been chosen to provide reference data for several forthcoming studies.

Our results suggest that the PUL2.0 can be used to follow changes of upper limb function over time. The scale appears to be more useful to detect shoulder and mid- domain changes while appears to be less sensitive in patients with function limited to the distal level. Because of the proximal to distal gradient of progression, however the changes are not linear and a wide variability can be observed in the whole cohort. The use of the entry levels as stratification criteria allows to identify more precise trajectories and to better define the expected progression over two years. This information will be valuable at the time of designing new studies or interpreting the results of the ongoing trials.

Further studies in larger cohorts are needed to further validate and explore the statistical properties of the PUL2.0, including the assessment of the Minimal Clinical Important Difference (MCID) that was not established in this cohort. Larger studies will also help to assess if and how contractures affect the level of performance.

## Supporting information

S1 File PUL 2.0 original scoresheet. (TIF)

## References

[1] Mercuri E, McDonald C, Mayhew A, Florence J, Mazzone E, Bianco F, et al International workshop on assessment of upper limb function in Duchenne Muscular Dystrophy: Rome, 15–16 February 2012. Neuromuscul Disord. 2012;22(11):1025–8. doi: 10.1016/j.nmd.2012.06.006 . 22795657 10.1016/j.nmd.2012.06.006 PMC3500683

[2] Mazzone ES, Vasco G, Palermo C, Bianco F, Galluccio C, Ricotti V, et al A critical review of functional assessment tools for upper limbs in Duchenne muscular dystrophy. Dev Med Child Neurol. 2012;54(10):879–85. doi: 10.1111/j.1469-8749.2012.04345.x . 22713125 10.1111/j.1469-8749.2012.04345.x

[3] Mayhew A, Mazzone ES, Eagle M, Duong T, Ash M, Decostre V, et al Development of the Performance of the Upper Limb module for Duchenne muscular dystrophy. Dev Med Child Neurol. 2013;55(11):1038–45. doi: 10.1111/dmcn.12213 . 23902233 10.1111/dmcn.12213

[4] Pane M, Mazzone ES, Fanelli L, De Sanctis R, Bianco F, Sivo S, et al Reliability of the Performance of Upper Limb assessment in Duchenne muscular dystrophy. Neuromuscul Disord. 2014;24(3):201–6. doi: 10.1016/j.nmd.2013.11.014 . 24440357 10.1016/j.nmd.2013.11.014

[5] Pane M, Mazzone ES, Sivo S, Fanelli L, De Sanctis R, D’Amico A, et al The 6 minute walk test and performance of upper limb in ambulant duchenne muscular dystrophy boys. PLoS Curr. 2014;6 doi: 10.1371/currents.md.a93d9904d57dcb08936f2ea89bca6fe6 . 25642376 10.1371/currents.md.a93d9904d57dcb08936f2ea89bca6fe6 PMC4208936

[6] Pane M, Fanelli L, Mazzone ES, Olivieri G, D’Amico A, Messina S, et al Benefits of glucocorticoids in non-ambulant boys/men with Duchenne muscular dystrophy: A multicentric longitudinal study using the Performance of Upper Limb test. Neuromuscul Disord. 2015;25(10):749–53. doi: 10.1016/j.nmd.2015.07.009 . 26248957 10.1016/j.nmd.2015.07.009 PMC4597096

