# Supplementary figures and images for: Upper limb function in Duchenne muscular dystrophy: 24 month longitudinal data
Source: PLoS One. 2018 Jun 20;13(6):e0199223. doi: 10.1371/journal.pone.0199223 (PMC6010252; doi:10.1371/journal.pone.0199223)

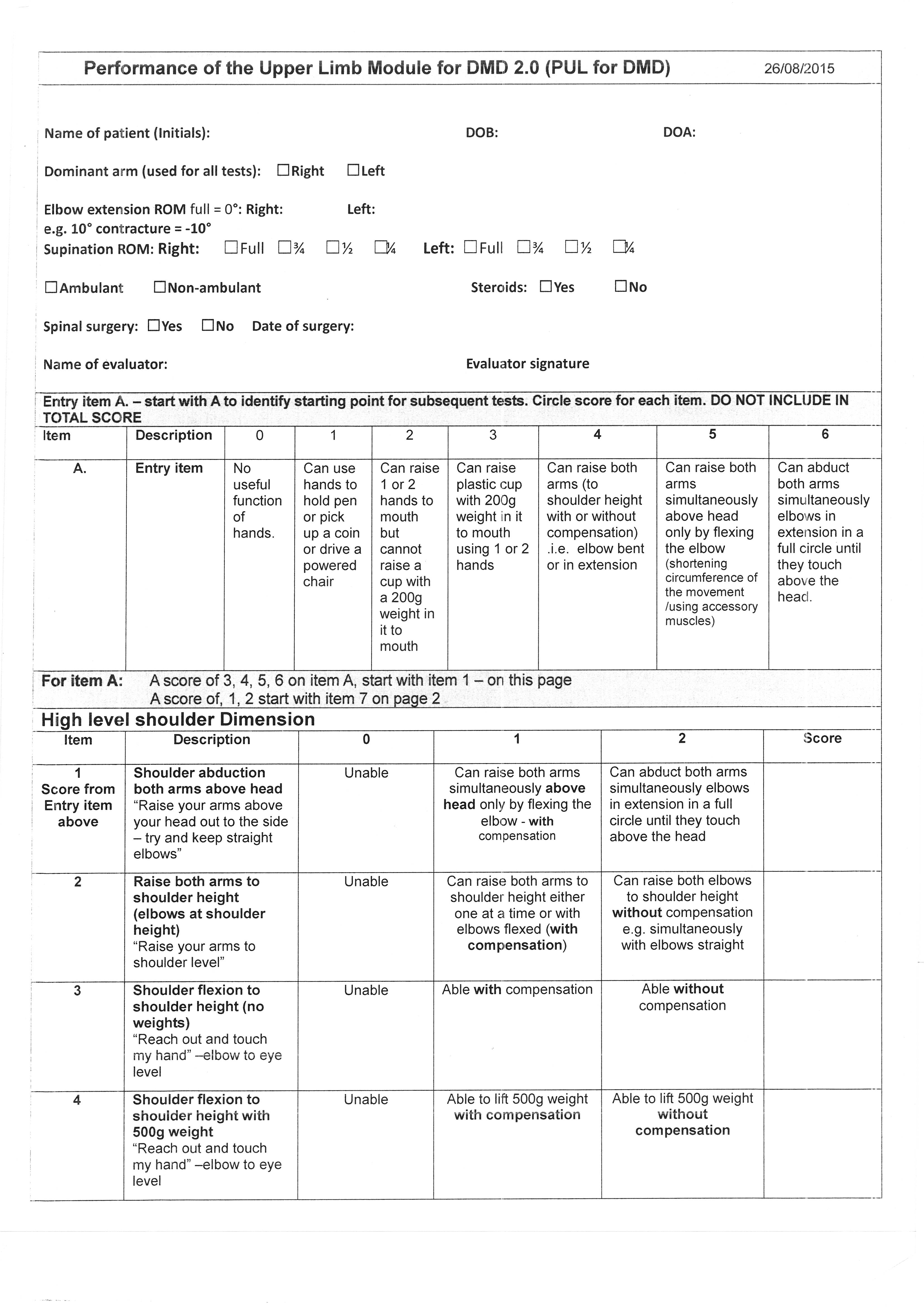

Supplement: S1 File — (TIF) [file pone.0199223.s001.tif]
